# Assessing the impact of a knowledge translation intervention on physical therapists’ self-efficacy and implementation of motor learning practice

**DOI:** 10.1186/s12909-023-04304-2

**Published:** 2023-05-23

**Authors:** Michal Kafri, Yasmin Levron, Osnat Atun-Einy

**Affiliations:** Department of Physical Therapy, Faculty of Social Welfare and Health Sciences, University of Haifa, Haifa, Israel

**Keywords:** Knowledge translation, Motor learning, Physical therapy, Skill acquisition, Professional education

## Abstract

**Background:**

The application of motor learning (ML) principles and research in physical therapy can optimize patient outcomes. However, the translation of the accumulated knowledge in ML to clinical practice is limited. Knowledge translation interventions, which are designed to promote changes in clinical behaviors, have the potential to address this implementation gap. We developed, implemented, and evaluated a knowledge translation intervention for ML implementation that focuses on building clinical capacity among physical therapists for the systematic application of ML knowledge in clinical practice.

**Methods:**

A total of 111 physical therapists underwent the intervention, which consisted of the following: (1) an interactive didactic 20-hour course; (2) an illustrated conceptual model of ML elements; and (3) a structured clinical-thinking form. Participants completed the Physical Therapists’ Perceptions of Motor Learning (PTP-ML) questionnaire pre and post intervention. The PTP-ML was used to assess ML-related self-efficacy and implementation. Participants also provided post-intervention feedback. A sub-sample (n = 25) provided follow-up feedback more than a year after the completion of the intervention. Pre–post and post-follow-up changes in the PTP-ML scores were calculated. The information gathered from the open-ended items of the post-intervention feedback was analyzed to identify emerging themes.

**Results:**

Comparing pre- and post-intervention scores, significant changes were found in the total questionnaire scores, self-efficacy subscale scores, reported implementation subscale scores (*P* < .0001), and general perceptions and work environment subscale score (*P* < .005). The mean changes in the total questionnaire and self-efficacy scores also significantly exceeded the Reliable Change Index. In the follow-up sample, these changes were maintained. Participants felt that the intervention helped them organize their knowledge in a structured manner and consciously link their practice elements to concepts in ML. Discussion of clinical cases was reported to be the most valuable educational method, and the illustrated conceptual model of ML elements was the least valued. Respondents also suggested support activities to maintain and enhance the learning experience, including on-site mentorship and hands-on experience.

**Conclusions:**

Findings support the positive effect of an educational tool, most prominently on physical therapists’ ML self-efficacy. The addition of practical modeling or ongoing educational support may enhance intervention effects.

**Supplementary Information:**

The online version contains supplementary material available at 10.1186/s12909-023-04304-2.

## Background

In physical therapy practice, motor learning (ML) is often a primary focus of treatment. ML refers to a set of internal processes associated with practice that leads to relatively permanent changes in the capability to perform a motor skill [1]. Despite widespread acceptance of the potential benefits of applying ML research in physical therapy practice, which can optimize the practice effect [2–9], implementation of ML knowledge in physical therapy practice lags behind the accumulated research [2, 9–11]. A delay in modifying clinical behaviors may be associated with various clinician-related factors, such clinicians’ knowledge, self-efficacy regarding the subject matter, perceptions regarding the significance of the knowledge for patients [12, 13], and domain-related factors such as the complexity of the knowledge [9] and organizational-related factors such as time resources and mentorship [14].

### Knowledge translation in healthcare

In healthcare, knowledge translation (KT) is the exchange, synthesis, and application of research findings in practice settings, with the aim of promoting changes in clinical routines based on research evidence [15]. The KT process includes several key components: identification of the problems in translating research knowledge into practice; knowledge development and selection; analysis of context; knowledge transfer interventions; and knowledge utilization, selection, and customization of implementation strategies [15–19].

Various KT implementation strategies can be used by medical and healthcare professionals to promote knowledge in practice. These strategies can be single or multifaceted, and range from conceptual frameworks or models, through educational activities, to audits, feedback, and outreach [20, 21]. Many current KT activities are based on continuing professional education such as courses and workshops [22–24]. In the healthcare field, multifaceted interventions that use an interactive educational format have been generally shown to have a greater effect on changing professional behavior than those using a single didactic format [24, 25]. Moreover, although educational courses that included only didactic sessions were recognized as a necessary first step in the process of change, they resulted in only a small-to-moderate improvement in professional practice [26] and smaller improvements in patient outcomes [22, 27]. It should be noted that many variables influence the effectiveness and consistency of KT interventions, including the type of activities, the target population, and the organizational (setting) contexts [28].

### Knowledge translation in the field of motor learning

Specifically in relation to ML, continuous efforts are required to advance the integration of ML knowledge into clinical practice. Research on KT strategies in ML remains largely limited. The few examples include the Delphi study, which generated a consensus for definitions of ML elements in relation to the clinical context [29, 30], and the development of the Motor Learning Strategies Rating Instrument, which aims to understand the use of ML strategies by physical therapists (PTs) [11, 31]. Another example is the development of conceptual frameworks such as the accelerated skill acquisition program [3]. A review of the conceptual frameworks reveals the factors that may pose challenges to implementation (e.g., lack of uniformity of elements and large diversity in the approaches used to present the elements) [32]. These challenges highlight the multi-step nature of the KT process, which requires not only the development of dissemination tools but also the use of deliberate tools that guide the clinician during actual implementation.

To conclude, the slow dissemination of motor learning knowledge in physical therapy practice has prompted the need to implement knowledge translation processes to promote change in clinical routines. To address this need, a multifaceted intervention specifically designed for PTs was developed and implemented. The intervention was focused on building PTs’ capacity for systematic application of ML knowledge in their practice. Herein, we report the results of the aforementioned process.

This study had the following aims:

- To evaluate the influence of a “KT-ML” intervention on ML-related self-efficacy, reported ML implementation, and general perceptions and work environment among certified PTs.
- To evaluate the long-term influence of the intervention on ML-related self-efficacy, reported ML implementation, and work environment in a subsample of certified PTs.
- To evaluate the process of change and assimilation immediately after the intervention and over time as perceived by the PTs.
- To estimate the intervention from the participants’ perspective.

## Methods

### Study design

This interventional study was conducted in a pre-intervention, post-intervention, and follow-up design with three assessment time points: time 1 (T1), pre- intervention; time 2 (T2), post (i.e. immediately after)-intervention; and time 3 (T3), in the follow-up phase. The main study population participated in the first two assessments, while a subsample also participated in the follow-up assessment. The study was approved by the ethics committee of the University of Haifa, and participants in both settings were informed that they could choose to opt out of the study at any time.

### Participants and setting

One-hundred thirty-five PTs participated in the “KT-ML” intervention in two different settings: academic and workplace. In the academic setting, participation was offered within a master’s degree program in physical therapy. There was no credit offered for participation, and students were informed that participation was voluntary and would have no implications for their course grades. In the workplace setting the intervention was delivered as a continuing education course. All PTs who underwent the intervention were invited to participate in the study. Of these, 111 agreed to participate and completed a questionnaire pre- intervention (T1) and post-intervention (T2), and their data were included in the pre–post analysis. The remaining 24 participants agreed to participate but did not complete the post-intervention questionnaire and therefore were not included. To recruit the follow-up sample, PTs who completed the pre and post questionnaires were invited via personal email or through a designated contact coordinator in their workplace to complete a follow-up questionnaire. Twenty-five participants agreed to participate and completed the follow-up questionnaire (T3) 1–3 years after completing the intervention, and their data were entered into the follow-up analysis. Inclusion criteria were license to practice physical therapy in Israel and participation in the intervention program.

The main sample came to represent the participants in the “KT-ML” intervention. Using an estimate according to Cochran’s formula (which is used to calculate the sample size for a desired level of precision in survey research) [33], for 5% error and 95% confidence interval, the required sample size is 100 participants. Our study sample contained 111 subjects and thus passed the calculated requirement. According to the Rosner formula [34], measurement of follow-up in the comparative section required a sample of 24 PTs. Rosner’s formula takes into account the same variables as Cochran’s formula, but also includes a correction factor to adjust for the reduced variability in the sample due to the smaller population size [34, 35]. The calculation was evaluated in terms of power 80%, and 95% confidence interval, taking into account the standard deviations demonstrated in the PTP-ML questionnaire [36]. Our sample contained 25 PTs and thus met the calculated requirement. Table 1 presents the personal and professional background of the samples. All participants provided written informed consent. Participants who completed the follow-up questionnaire signed an additional consent form specifically for the follow-up.

**Table 1:** Demographic and professional background of the respondents

Characteristic variables	Pre-post sample(N = 111)	Follow-up sample (N = 25)
**Age, y, mean±SD (range)**	35.0 ± 7.5 (25-59)	37.5 ± 5.8 (29-42)
**Gender, %**	Female 79.4%	Female 85%
**Highest degree, %**		
Bachelor, Master, PhD	84.1%, 14%, 1.9%	86.4%, 9%, 4.5%
**Years of experience, mean±SD (range)**	8.5 ± 7.8 (0-33)	11 ± 6.7 (2-30)
**Employment, %**		
Full-time or more	16.3%	13.5%
Between full-time and half-time	76%	82%
Half-time or less	7.7%	4.5%
**Main field of practice, %**		
Neurology and orthopedic	29.2%	18.5%
Orthopedic	14.2%	7.4%
Neurology	26.4%	44.4%
Pediatrics	9.4%	11.1%
Other	11.3%	11.1%
Mixed	9.4%	7.4%
**Main work setting, %**		
Orthopedic outpatients	14%	14.8%
Rehabilitation center	55.1%	66.7%
General hospital	12.1%	0%
Pediatric settings	6.5%	7.4%
Other	12.1%	11.1%

SD, standard deviation

### The “KT-ML” intervention

#### Description of the intervention

**Fig. 1:**
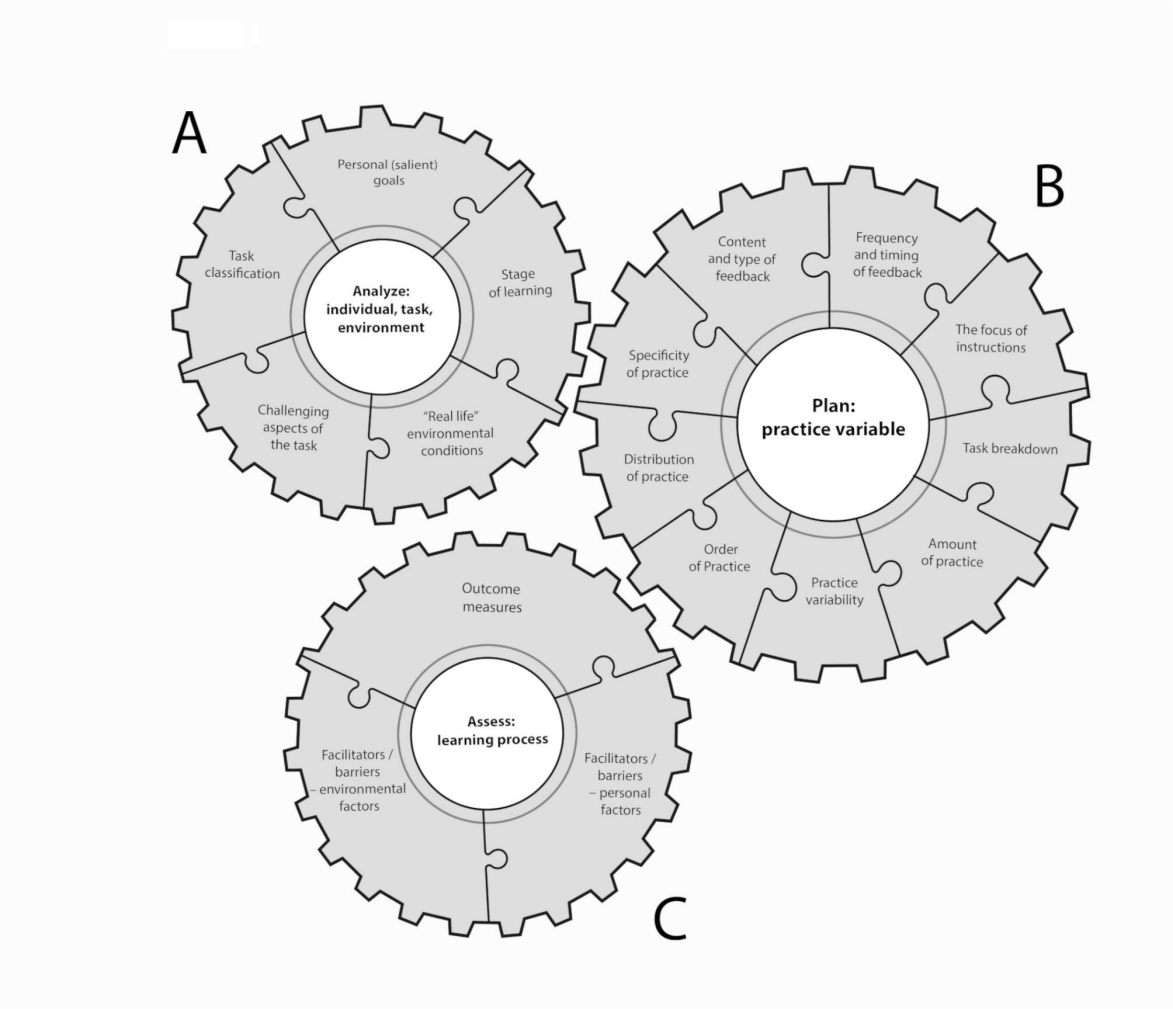
An illustrated conceptual model of motor learning elements The three inter-related cogwheels represent a different component of the ML intervention. The cogwheel representation emphasizes the interrelationships between the different parts of the model and its importance in the advancement of the learning process

**The “KT-ML” intervention** was designed to emphasize a comprehensive, systematic implementation of ML elements in PT practice and developed for PTs with basic knowledge in ML. The intervention was multifaceted and consisted of the following components:

1. **The didactic module** included an interactive 20-hour course composed of an introductory presentation of the general concepts in ML, practice variables, and learning strategies [32, 37]. The content was delivered using multiple educational strategies. A detailed description of the module is presented in *Additional file 1*. Participants received handouts of the presentations, a list of selected references and links to relevant educational resources. A PhD-level physical therapist and motor learning researcher delivered the module.
2. **An illustrated conceptual model of ML elements***(see* Fig. 1*)*:

The illustrated conceptual model of ML elements comprises three inter-related cogwheels representing different components of the ML intervention. The innovatively designed model provides practical guidance on the “how-to” of implementing motor learning-based practice in a clinically meaningful way. The model was developed on the basis of literature in the field of motor control and learning. [8, 38–42]

Cogwheel A “Analyze: individual, task, environment” includes elements related to skill acquisition. Completing the elements in this cogwheel enables the clinician to design a patient-centered practice and is the starting point for a practice plan. Cogwheel B “Plan: practice variables” includes the procedural aspects of practice (i.e., practice variables) that must be adjusted in reference to the data gathered in the first cogwheel (e.g., feedback frequency is determined on the basis of the learning stage). Cogwheel C “Assess: learning process” addresses elements of learning assessment.

The cogwheel representation clarifies the interrelationships between the different parts of the model and its importance in the advancement of the learning process. The optimal operation of the system, that is, an efficient learning process, depends on the specification of each element relative to itself and the specifications of the other elements. The specification of each item is dynamic and requires continuous refinement based on the outcomes and clinical judgment of the practitioner.

***3.*Structured clinical-thinking form***(see Additional file 2)*:

A structured form was used to guide the clinical thinking, planning, and clinical decision-making required for ML intervention. This form outlines the practical steps required to implement the conceptual model.

### Intervention development

**Fig. 2:**
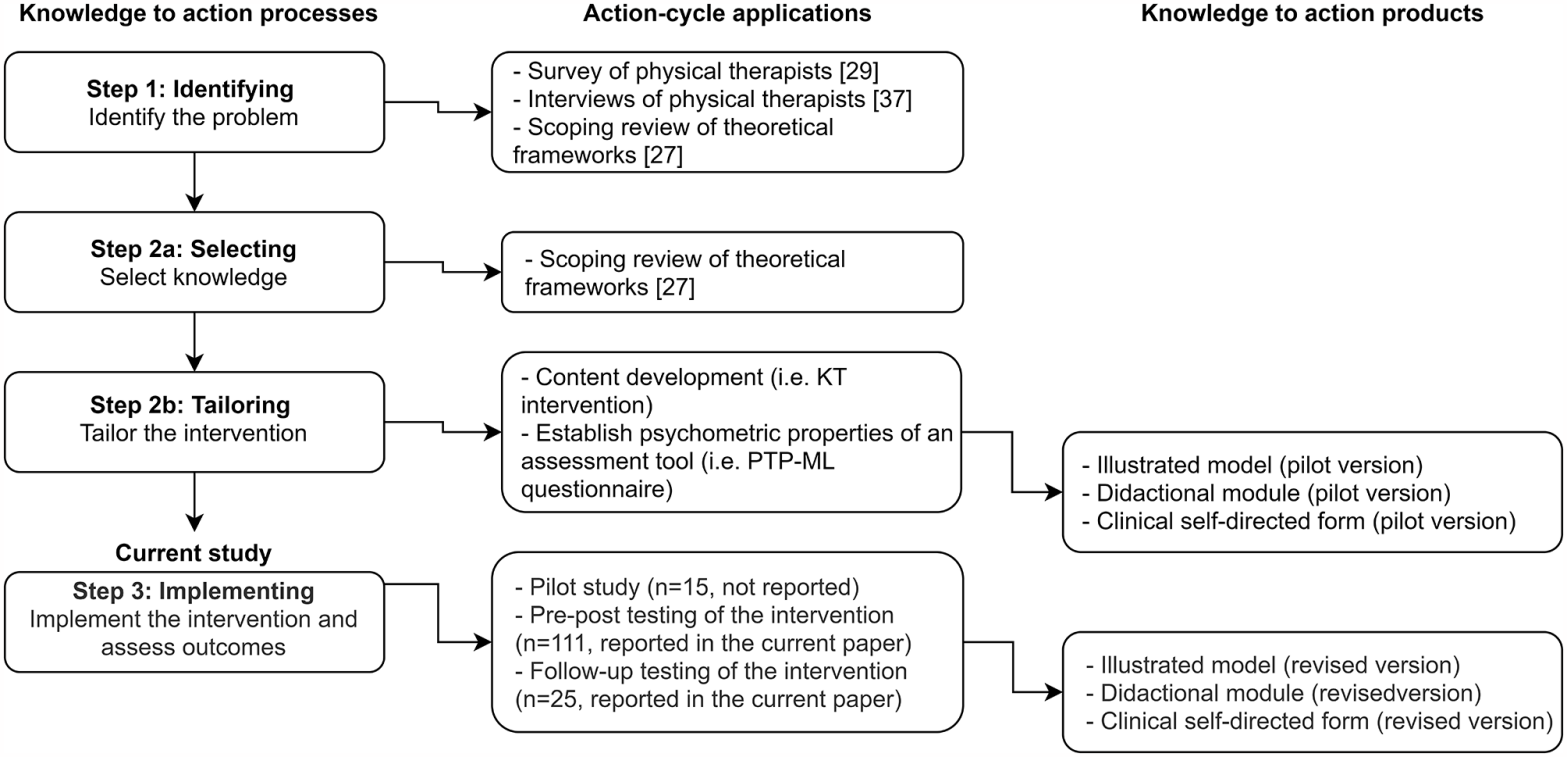
The intervention development process PTP-ML, Physical Therapists’ Perceptions of Motor Learning; KT, knowledge translation

The development of the intervention was guided by the core elements of the KT process [2–9]. Development proceeded in three steps: (1) identifying the problems in translating research knowledge to practice, (2) selecting and tailoring the intervention, and (3) implementing the intervention. The development process which included these three steps is presented in a flow chart (Fig. 2). Steps 1 and 2 were conducted prior to this study and are reported elsewhere [32, 36, 43]. This study presents the results of the implementation step. In step 1, the challenges in and barriers for translating ML knowledge into practice were identified via a practitioners’ survey [36], practitioners’ semi-structured interviews with practitioners [43] and a literature review [32]. Based on the identified barriers, we recognized the need for a more systematic approach to promote the implementation of ML elements. Accordingly, in step 2 we selected knowledge of motor learning elements applicable to physical therapy including theoretical concepts, practice variables and learning strategies [32]. In line with this knowledge as a second phase within step 2 we tailored the intervention first by developing a conceptual model that assembles the elements of ML into a coherent structure, and then by developing a tools for didactic intervention. In step 3, a pilot intervention was conducted in a small sample of PTs (n = 15); their feedback was used to modify the intervention. Modifications included increasing the interactive elements of the process and providing more extensive guidance for implementation. In addition, we made changes to the clinical-structured clinical thinking form. Finally, the intervention was implemented on a larger sample and assessed as reported below.

### Procedures and instruments

#### Procedures

Participants completed the questionnaire at the first and last meetings of the intervention. In the last meeting they also completed post-intervention feedback. A subsample of the participants completed the questionnaire and the follow-up feedback form on the follow-up occasion.

### Instruments

#### a. physical therapists’ perceptions of Motor Learning (PTP-ML) questionnaire

ML-related self-efficacy and implementation were assessed by using the PTs’ perceptions with the PTP-ML questionnaire. Construct validity, internal consistency, and test–retest reliability of this questionnaire have been shown to be satisfactory [10]. This self-report questionnaire encompasses three subscales. The “ML self-efficacy” subscale (12 items) measures respondents’ confidence in their knowledge and ability to explain ML principles or terms. The “implementation” subscale (12 items) measures self-reported implementation of ML principles, and the “general perceptions and work environment” subscale (4 items) measures environmental factors that may support knowledge application.

Items are rated on a 5-point Likert-type scale ranging from “strongly agree” to “strongly disagree” in the “self-efficacy” subscale or from “very little” to “very much” in the “implementation” and “general perceptions and work environment” subscales. The “implementation” subscale contains an additional option to check “unaware of this ML principle” to indicate that the respondent was unaware of the concept in question. For further details about the tool, see the study by Atun-Einy and Kafri [36]. The score of each subscale is the mean of all items in the subscale, and the total score is the mean of all items. Higher scores indicate higher ML self-efficacy and implementation and the presence of more enablers of ML implementation in the workplace.

#### b. post-intervention feedback

The post-intervention feedback consisted of nine statements concerning satisfaction with the intervention, changes in practice, and the usability of the intervention. Items were rated on a 5-point Likert scale ranging from 1 (*do not agree with the statement*) to 5 (agree to a large extent). These statements are listed in Table 2.

**Table 2:** Distribution of responses in the post-intervention feedback

Statement	High level agreement	Agree	Disagree
I would recommend participating in the educational intervention to other colleagues	72.2%	25%	2.8%
**Change in my practice**			
I am able to apply the knowledge I gained from the model to my work	60.2%	36.1%	3.7%
Use of the model makes me spend more time planning treatment	49.1%	45.4%	5.6%
Use of the model led to a change in the way I provide treatment	31.5%	50%	18.6%
**Usability**			
Physiotherapists need instruction and training to use the model	69.4%	28.7%	1.9%
Understanding the model only enriches my knowledge (reversed item)	2.8%	8.3%	88.9%
The model is too difficult to implement (reversed item)	3.7%	10.2%	86.1%
Only clinicians with several years of experience can make use of the model (reversed item)	5.6%	8.3%	86.1%

To obtain supplementary insights and encourage the respondent to give a rich description using qualitative content, the post-intervention feedback also included five open-ended questions about the application experience (what did you learn from the course or what was new for you? What variables in your practice do you currently consider and had not considered before the intervention? Which of the concepts presented in the intervention were simple to apply? Which of the concepts presented in the intervention were difficult to apply? Are there any theoretical concepts whose application is particularly challenging?).

#### c. follow-up feedback

The follow-up feedback assessments included nine statements concerning long-term changes in participants’ clinical practices and the perceived educational value of the educational methods (rated from high to insignificant).

### Data analysis

To compare the pre- and post and the post and follow-up questionnaire scores, a 2 × 2 analyses of variance (ANOVA) using a mixed repeated-measures design with one within-subject variable (time point) and one between-subject variable (intervention setting: academic, workplace) were performed for each subscale and the total score. Changes in the frequency of the “not aware” rating in the implementation subscale were reported using descriptive statistics.

The reliable change index (RCI) was used to estimate changes in scores that exceeded the test-retest coefficients previously calculated using the PTP-ML questionnaire [36]. The RCI is a statistical method used to determine whether a change in scores over time, such as after an intervention, is statistically significant and beyond the expected range of measurement error. It considers the variability of measure scores and the measurement error associated with the measure used. The RCI is calculated by comparing the difference between a person’s pre-intervention and post-intervention scores to the expected amount of change due to measurement error. If the change in scores exceeds the expected range of measurement error as determined by the RCI, the change is considered reliable. The RCI is useful for assessing the effectiveness of an intervention and for evaluating individual changes in scores.

The RCI was computed for the group and for each participant, and a Z threshold of 1.96 was used to determine its significance. Individual RCI scores enable evaluation of the direction of individual changes as increased, decreased, or stable. RCI data were reported for the group, as well as the frequency of respondents who passed the RCI threshold.

Pearson or Chi-square correlations according to the variable type were used to analyze correlations between the changes in the questionnaire total and subscale scores and the demographic variables, including gender, age, years of experience, academic degree, and main field of practice. Data were analyzed using SPSS 23 software. Significance threshold was set at 0.05.

The responses to the open-ended questions were analyzed using content analysis methods that allow identification of themes that emerged in the particippants’ responses [44]. Coding was performed by one of the authors (YL) and was reviewed by a second author (OAE) to ensure that the interpretations were valid and reliable.

## Results

### Effects of the intervention according to the PTP-ML questionnaire

The pre-intervention (T1) and post-intervention (T2) questionnaire scores are presented in Table 3.

**Table 3:** Analysis of pre–post-intervention questionnaire scores

Variable	Pre-intervention (T1)	Post- intervention (T2)	Intervention effect (pre-pot)	Effect size	RCI	RCI: Subjects distribution	Between and interaction effects
	Mean (standard deviation) or median (inter-quartile range)			partial η^2^		Number of participants with: An increase (%) A decrease Minor change	
Self-efficacy	2.73 (0.52)	3.90 (0.45)	F_(1,109)_ = 435 p < .0001	0.66	3.07*	89 (80.2%) 0 22	NS and NS
Reported ML implementation	3.02 (0.50)	3.48 (0.42)	F_(1,109)_ = 69 p < .0001	0.24	1.60	46 (41.4%) 0 65	NS and NS
General perceptions and work environment	2.75 (2.0-3.25)	3.0 (2.25–3.5)	F_(1,108)_ = 13 p < .0005	0.06	0.67	24 (21.8%) 3 84	F_(1,108)_ = 8.67, p < .004, partial η2 = 0.038 and NS
Total	2.88 (0.45)	3.69 (0.37)	F_(1,109)_ = 289 p < .0001	0.57	2.75*	77 (69.4%) 0 34	NS and NS

RCI, Reliable change index

*Reliable change index > Z

***Total questionnaire score***: Pre-post comparisons of the total score showed a significant increase in participants’ mean score following training: F_(1,109)_ = 289, p < .0001, effect size (η^2^) = 0.57. The mean group RCI was significant, with 69.4% of participants showing an increase in their total score. There were no group (i.e. setting) or interactions effects.

***Self-efficacy subscale score***: Pre–post comparison of the self-efficacy subscale score showed a significant increase in participants’ mean score following training: F_(1,109)_ = 435, p < .0001, effect size η^2^ = 0.66. The mean group RCI was significant, with 80% of participants showing an increase in self-efficacy. There were no group (i.e. setting) or interactions effects.

***Reported implementation subscale score***: Pre-post comparisons of reported ML implementation score showed a significant increase in participants’ mean score following training: F_(1,109)_ = 69, p < .0001, effect size η^2^ = 0.24. The mean group RCI did not reach significance, although 41% of the participants showed an increase in reported ML implementation. The reported implementation subscale of the questionnaire demonstrated a notable decrease in the frequency of the response “not aware”, which was particularly evident in three questionnaire items. In item B3 (“To what degree do you plan whether to give instructions on external focus of attention or on internal focus of attention?“), the frequency of “not aware” response decreased from 17 to 0%. Similarly, in item B6 (“To what extent do you plan whether the feedback you give will be based on knowledge of results or knowledge of performance?“), the frequency decreased from 23 to 0.9%. Finally, in item B10 (“To what extent do you include positive reinforcement (reward) in the learning process?“), the frequency of “not aware” response decreased from 13.5 to 0.9%. There were no group (i.e. setting) or interactions effects.

***General perceptions and work environment subscale score***: There was a small but significant increase in participants’ mean score following training: F_(1,108)_ = 13, p < .0005, effect size η^2^ = 0.06. The mean group RCI did not reach significance, although 22% of the participants showed an increase in this subscale. There was a small but significant group difference (F_(1,108)_ = 8.67, p < .004, partial η^2^ = 0.038); so that the score of the group that underwent the intervention in the clinical setting was lower across measurement time points. No interactions were observed.

#### Follow-up changes

There were no significant differences in questionnaire subscales and total scores between the post-intervention and follow-up measurements (self-efficacy scale [mean post-intervention, 3.94 (0.45); mean follow-up, 3.74 (0.5); F_(1,20)_ = 1.12, p = .3]; reported ML implementation scale [mean post-intervention, 3.64 (0.47); mean follow-up, 3.59 (0.41); F_(1,20)_ = 0.2, p = .65]; general perceptions and work environment in ML scale [mean post-intervention, 3.05 (0.56); mean follow-up, 3.15 (0.53); F_(1,20)_ = 0.05, p = .81], and total score [mean post-intervention, 3.79 (0.40); mean follow-up, 3.66 (0.41); F_(1,20)_ = 0.69, p = .41]), indicating that the change in scores between pre-and post-intervention was maintained in the long term. There were no setting differences or interactions.

### The effect of background variables

No significant correlations were found in the pre-post changes in questionnaire scores and demographic variables, including gender, age, years of experience, academic degree, and main field of practice.

### Post-intervention feedback

The structured feedback included closed and open-ended questions. All participants completed the closed questions, and 87% completed the open-ended question. Table 2 shows the respondents’ agreement with post-intervention feedback items. The vast majority of participants (97%) said that they would recommend the intervention to other colleagues. For the three items referring to changes in practice, most of the respondents indicated that they were able to clinically apply the knowledge to their practice and that after participating in the intervention they spent more time planning their treatment. Agreement on a change in practice was somewhat lower, and 18.6% did not indicate that they had made a change in their practice after participating in the intervention. Participants found the knowledge easy to implement, and believed that no previous experience was needed in order to participate in the intervention.

### Open-ended questions

The content analysis of the responses to the open-ended questions of post-intervention feedback items revealed three themes. These themes reflect the participants’ experiences and thoughts regarding the intervention and how their experiences were related to the benefits of the intervention. Several themes included two to five subcategories. Each theme is presented followed by a quote designed to provide grounding for the theme in the data [13] (Note that the reference to the participants’ number, presented at the end of each quote, does not reflect the sample size because the numbers were originated from a previous study. [36]).

### Gaining a systematic framework to organize and structure ML knowledge

Participants felt that the intervention helped them to organize their knowledge in a structured manner. This in turn, allowed them to gain a better understanding of the new knowledge in an appropriate context and to link old and new knowledge. Participants felt that the organization of knowledge improved their grasp of both the breadth and the boundaries of ML knowledge.

“It arranges my thought, how to move forward, and allows me to focus on those points that I need to improve and focus on” (participant 26), “The intervention is very structured; it creates order in the chaos.” (participant 306).

### Bi-lateral integration of knowledge and practice

Participants felt that they were able to consciously link their practice elements to concepts in ML. They considered the intervention as an assistive strategy to apply knowledge in practice, and it encouraged them to link their practice back into its theoretical foundations.

“It allowed me to do a process of integration, which I did not have before, and to bring the principles to a much higher level of implementation than I have done so far.” (participant 53).

“It gave me a variety of techniques and a solid foundation that I could rely on in my treatments.” (participant 171).

“I gained a great deal of knowledge and received a more detailed explanation of concepts I already knew … a connection between work I used to do and concepts in ML” (participant 330).

“ Suddenly, my behavior and that of my patients became more understandable to me.“ (participant 16).

### Specific changes in clinical practices

Participants elaborated on the change in their practices in several aspects.

a. Expanded a priori planning phase of the intervention.

“Nowadays, I know that before treatment, you need to plan it - something I have neglected over the years.” (participant 314).

b. Adding outcome measures.

“I am currently planning more of the outcome measures that I will use to assess the patients’ learning process.“ (participant 329).

“The model required me to focus on a specific goal and to measure my progress.” (participant 36).

c. Reduction of clinical behaviors that did not match theoretical knowledge.

“To give correct and optimal feedback to the patient at the appropriate time—much less talking !!” (participant 26).

d. Informed choices of practice parameters.

“Adjusting levels of difficulty for the learner at various stages … to map at what stage of learning the patient is.” (participant 230).

e. Specific new emphasis: Participants gave specific examples of changes in their clinical behavior.

“Choosing skills and personal goals, creating motivation” (participant 241.

“Creating a degree of diversity that will be meaningful for improving the learning process” (participant 338).

“More challenges in treatment.“ (participant 334).

### Follow-up feedback

Participants in the follow-up phase (n = 25) completed the follow-up feedback form in addition to the “PTP-ML” questionnaire (findings detailed above). Table 4 presents the results of the closed-ended items in the follow-up feedback.

**Table 4:** Intervention follow-up feedback

Statement	High level agreement	Agree	Disagree
As a physical therapist, I benefited from the educational program.	80%	20%	0%
My patients benefited from the educational program.	70%	23.3%	6.7%
The KT-ML intervention led to change in the way I provide treatment.	43.4%	46.7%	10%
There are treatment options I abandoned as a result of the program.	6.7%	30%	63.3%
The training outputs were easily integrated into my clinical practice.	46.7%	46.7%	6.7%
The educational program led to change in the way I provide treatment.	43.4%	46.7%	10%
Self-processing was needed to apply the knowledge I gained.	63.3%	30%	6.7%
I am interested in serving as a change broker in my workplace to promote the use of motor-learning knowledge in practice.	53.3%	30%	16.7%
I would like to have attend a continuing follow-up workshop on the topic.	70%	23.3%	6.7%

When asked about the feasibility of implementation of the intervention for their patients, 56.7%, 26.7%, and 16.7% of the respondents reported that implementation was feasible with most, half, and few of their patients, respectively.

Table 5 presents participants’ evaluation of educational methods’ contribution. Discussion of clinical cases was the most valuable method, and the conceptual model of ML elements was the least valued. Respondents also suggested support activities to enhance the learning experience, including on-site mentorship and team journal clubs.

**Table 5:** Reported value of the educational methods

Educational methods	High level of value	Medium level of value	Insignificant value
Discussion of case studies	96.7%	3.3%	0%
Self-experiencing using the clinical thinking form to apply ML-based practice to their patients	96.4%	3.6%	0%
Practical illustration of motor learning elements	89.7%	10.3%	0%
Presentation of research articles	51.7%	34.5%	13.8%
A structured clinical thinking form	53.6%	25%	21.4%
Illustrated conceptual model of motor learning elements	30.8%	15.4%	53.8%

When asked about the impact of the intervention over time, the majority of respondents (58.6%) reported that the greatest change occurred two months after the intervention. A total of 10.3% reported that the greatest change occurred immediately after the intervention. A total of 17.2% reported that the greatest change occurred several months after the intervention. and 6.9% reported that the greatest change occurred after one year. Of all respondents, 6.9% reported that no major change occurred after the intervention. In a note to this item, six participants stated that their awareness of the topic was greatest immediately after completing the intervention, and that implementation subsequently diminished.

## Discussion

This study reports the short- and long-term effects of a newly developed “KT-ML” intervention on PTs’ self-efficacy and reported ML implementation. The evaluation was conducted using a psychometrically sound outcome measurement tool. Our results support the usefulness of the intervention.

### Changes in self-efficacy toward motor learning

Pre–post changes in self-efficacy revealed a robust post-intervention increase. This was indicated by the large effect size and the fact that the effect stood the test of RCI. This effect was maintained over the long term, as indicated by follow-up testing. The pre-intervention self-efficacy score is similar to the level of self-efficacy reported by PTs in Israel and Brazil [36, 37] This may support the generalizability of the findings.

It is difficult to reconcile the consistent finding of PTs’ low-to-moderate self-efficacy with respect to ML [9, 45], given the sweeping recognition of ML as an important and integral part of physical therapy practice [36, 43, 45]. Low self-efficacy was shown to impede translation of positive attitudes into everyday clinical behaviors [12, 13, 46]. Increasing self-efficacy, although not a sufficient dimension for determining actual behavioral change, is important for increasing the likelihood of translating knowledge change into practice. Previous studies have identified self-efficacy as a meaningful outcome in professional training studies [47–49]. The mechanism underlying the effects of self-efficacy on behavior has been linked to cognitive motivational factors. In the context of ML, Levac et al. [9] tested the effect of a KT intervention on the application of ML in VR training. Their KT intervention incorporated five components among which were an interactive e-learning module, hands-on learning, and mentorship. Similar to our findings, they reported an increase in participants’ confidence in the use of ML strategies [9].

### Changes in reported implementation

In the present study, the ML implementation subscale score increased significantly after participation in the intervention, and this change was maintained over the long term. Importantly, there was a decrease in the number of respondents who reported that they were “not aware” of an item. This suggests that after the intervention, the scope of the participants’ thoughts about their clinical practice widened. For example, after the intervention, no respondent reported being “not aware” of the question about planning the type of instructions, relative to 17% before the interventions, meaning that respondents gained new knowledge about the categorization of instructions and were able to implement this knowledge into their clinical decision-making.

The changes in the implementation subscale were less robust relative to those in the self-efficacy subscale (i.e., moderate relative to large effect size; 40% passed the RCI relative to 80%). The level of self-reported implementation post-intervention was maintained in the long term. The gap between self-efficacy and implementation indicates that changing actual clinical behavior is a complex process, which probably involves the entire healthcare context (e.g., organizational factors) [19]. In a study that evaluated an occupational therapy mentorship program, self-efficacy and clinical performance were found to be correlated. The nature and direction of the association was, however, individual, and changes in self-efficacy and performance did not always occur at the same rate [49]. In another study, Levac et al. [9] tested changes in confidence as well as in actual implementation of ML elements by rating video-recorded virtual reality-based ML sessions. They found a greater increase in confidence that did not fully translate to a behavioral change (i.e., actual implementation). One may assume that change occurs in a dynamic reciprocal process, in which change in self-efficacy usually precedes change in implementation.

### Changes in general perceptions and work environment

There was a significant increase in the mean score for general attitudes and perceptions about the workplace environment following training, but with a small effect size. In addition, the RCI test demonstrated that most participants did not experience a reliable positive change in this subscale. This finding is expected since the intervention focused on the individual physical therapist and their practice, not on the workplace or organizational levels.

### Setting-based and professional background differences

The intervention was delivered in two educational contexts: academic and workplace settings. No preferable training setting could be identified. Contrary to our findings, some studies postulate that changes in clinical behavior are more likely when clinical education occurs on-site. For example, it was found that local knowledge brokers facilitate behavioral changes, such as the uptake of measurement tools [50]. It is possible that on-site programs have an advantage if they include organizational components that support individual-level changes [51]. Our intervention did not include this aspect.

The effect of the intervention did not differ as a function of professional background factors, such as field of practice or years of experience. It is possible that the basic concepts taught in the intervention were not field-specific and therefore relatable to PTs from all fields of practice. Regarding years of experience, there may be a tradeoff between greater experience and more current knowledge in the field.

### Long-term effects

The effectiveness of the intervention is further supported by the long-term maintenance of changes in all subscales. Prior studies stressed the need for long-term follow-up to test the effect of KT interventions [52], but current studies in physiotherapy or health professions usually lack this component. The long-term maintenance of the intervention effects should be interpreted with caution as the follow-up phase only included 23% of participants, and it is not possible to rule out that the follow-up participants had a more positive attitude toward the intervention.

### Participants evaluation of the intervention

The immediate feedback at the end of the intervention and the feedback given by a sub-sample later in the follow-up assessment provided insights about the feasibility, applicability, and aspects of change following the “KT-ML” intervention. Feedback showed that respondents were highly satisfied with the intervention and positively evaluated its clinical contribution, which occurred mostly immediately or soon after the intervention was completed. Participants also recognized the intervention as a useful clinical tool for promoting ML-based practice. It should be noted that participants expressed the need for further training, including more hands-on experience. The PTs proposed on-site mentorship with actual cases as an additional support activity. This would allow learners to directly experience the complexities of real-world situations and capture subtleties that cannot be learned through other methods. For example, progression of task difficulty is influenced by the actual performance of the task, and the calibration of this process can only be practiced in real life experience with patients [53, 54]. The expressed need for multiple support activities may reflect the challenges of ML for PTs [43].

Participants recognized the practical contribution of the knowledge and reported changes in their treatment planning practices. The reported change in a priori planning of treatments is significant, since this component is repeatedly reported to be lacking and was identified by PTs as a barrier for ML practice [36, 43].

Participants indicated that the delivery of the intervention was suitable for PTs in all fields of practice and levels of experience, although explicit training guided by an instructor is necessary (as was delivered in the study). They thought that it was not too difficult to apply this knowledge in practice.

### Enablers of the intervention effects

We suggest two primary reasons for the immediate and long-lasting positive effects of the intervention. First, its development was based on a knowledge-to-action cycle. For example, we identified barriers in translating research knowledge in practice, such as the complexity of the field of knowledge and the lack of a framework that will lead to clinical thinking. Second, the intervention includes different formats that facilitate clinical use of the knowledge.

Complexity and ambiguity hinder the translation of ideas into clinical practice, as indicated by the literature and PTs themselves [36, 43, 55, 56]. On the basis of this understanding, our intervention systematically described the various elements of the ML field and offered a framework that binds them together (i.e., the cogwheel illustration model).

The evidence supports KT programs that encompass multiple formats (facets). Of the various methods employed in the intervention, the most highly valued method by the participants as aids to the learning process included discussions of clinical cases, presentation of empirical studies, and practical explanations of each ML element. These findings support the premise that effective training benefits from interactive education through real case studies [18, 57]. The use of real case studies for learners can be more effective than simple didactic training since it provides a variety of real-life examples and provides a context rather than simply relying on a verbal mode. The high rating also speaks to PTs’ interest in the pragmatic aspects of practice.

Another valued educational method was the “ML structured clinical thinking form,” which most respondents evaluated as a helpful means that facilitated and organized the decision-making process, explicitly directed practitioners’ attention to each ML element [15, 58], and assisted with the planning of treatment sessions. Participants in the intervention had hands-on experience with the application of the form on their own patients, which may have improved their confidence in its implementation. Such active experiences, in addition to the intensity of the intervention, has been shown to improve clinical practice in previous studies [37, 57, 59].

### Practice implications

The results suggest that the reported intervention is associated with important gains in PTs’ self-efficacy, and ML implementation, especially in the highly valued facets, may be routinely adopted in continued in-service PT education.

To further support the long-lasting changes demonstrated immediately after the intervention, we recommend conducting continuous training that is targeted to specific clinical contexts. This can be done by discussion and demonstration of clinical cases and may be embedded in the clinical setting. Implementation studies support the need for ongoing education to support long-term changes in clinical behavior. For example, PTs who participated in a 2-day neck pain management course and ongoing educational sessions showed significantly better results in managing their patients’ neck symptoms relative to PTs who participated in a 2-day course without additional educational support. This finding also demonstrates that optimization of educational interventions translates into better clinical outcomes. Local mentors and change agents represent another means for this purpose, since they create opportunities for and share the responsibility of changes in clinical behaviors.

Specifically, studies on the implementation of evidence-based practices have demonstrated the contribution of local change agents to the process of change [60–63].

### Limitations

Several limitations must be considered when interpreting the implications of our study. First, in terms of study design, we used an uncontrolled design that reflected the preliminary phase of the intervention. The absence of a control group is common in educational intervention studies. The use of RCI, which specifically demonstrated that the changes were beyond the change expected over time due to repeated exposure to the measurement tool, and the follow-up assessment that demonstrated persistent change strengthened the findings.

Second, the effect of the intervention was based on self-reports and mirrored respondents’ points of view. Since changes in healthcare professionals are a complex issue, and the effect of knowledge on behavior is limited [36], we cannot evaluate the practical application of this knowledge in the clinical setting. This study focused on practitioner-level changes, while the organizational context was not examined. Third, the study was not aimed at measuring changes in patients’ clinical outcomes. Evaluation of patient outcomes should be considered as a next step in an evaluation of the intervention. In this context, it is worth noting that although the conceptual model of the ML elements presented in the intervention includes elements aimed at facilitating patient-centered care, such as setting goals and specifying learning variables according to the stage of learning or skill type, it does not explicitly incorporate the behavioral aspects of training that are crucial for patient empowerment and self-management support, such as teaching problem-solving skills and educating patients about the effects of practice and exercise [64]. These aspects are essential for achieving preferable outcomes. Therefore, clinical expertise for delivering these components of training is expected across all fields in physical therapy [65, 66] and should not be overlooked when implementing motor learning-based treatment.

Finally, our follow-up findings were based on a smaller sample than the general sample.

## Conclusions

This study provided knowledge on KT in the field of ML in continued physical therapy education. The findings support the positive effect of such an educational tool, which is most prominent on the PTs’ ML self-efficacy. The addition of practical modeling, in which the instructor demonstrates the implementation of ML in treatments, may enhance the intervention effects. Continuous ongoing educational support is another way to promote the use of the knowledge gained in the intervention and changes in clinical behaviors.

## Electronic supplementary material

Below is the link to the electronic supplementary material.

Supplementary Material 1

Supplementary Material 2

## Data Availability

The datasets generated and/or analysed during the current study are available in the Zonedo repository, 10.5281/zenodo.7582167.

## Abbreviations

KT: Knowledge translation
ML: Motor learning
PTs: Physical therapists
PTP-ML: Physical Therapists’ Perceptions of Motor Learning
RCI: Reliable change index
SD: Standard deviation
