# Supplementary material for: Assessing the impact of a knowledge translation intervention on physical therapists’ self-efficacy and implementation of motor learning practice
Source: BMC Med Educ. 2023 May 23;23:369. doi: 10.1186/s12909-023-04304-2 (PMC10207706; doi:10.1186/s12909-023-04304-2)
Supplement: Supplementary file 1 — Supplementary Material 1 [file 12909_2023_4304_MOESM1_ESM.docx]

**Additional file 1. Didactic module structure and content**

| **Intervention outcomes** | |
| --- | --- |
| Learning outcomes | Plan and execute motor skill acquisition program applicable to patients in physical therapy. |
| **Educational module content** | |
| Introduction: definition of motor learning, performance versus motor learning, retention and transfer, motor learning theories, specificity of practice and generalizability, performance measures to track learning. | |
| Basic concepts: Stages of motor skill acquisition, goal setting, active involvement, problem solving, challenge, learning mechanisms: implicit or explicit, classification of motor skills | |
| Practice variables   - Instructions - Modelling - Practice variables: dose; task breakdown: simplification, segmentation and fractionization; order of practice: Blocked, serial and random; practice variability: constant and varied; distribution of practice: Massed and distributed. - Feedback: Internal and augmented; augmented feedback types: knowledge of performance and knowledge of results; timing; frequency and fading schedules: summary, bandwidth, average, self-controlled. | |
| Learning strategies   - Task specific; mental practice; observational learning; errorless learning; analogy learning; trial and error learning; dual-task learning; discovery learning. | |
| **Educational strategies** | |
| Didactic module | Describe the motor learning elements detailed above. |
| Experimenting with implementation. | During the practice the participants experimented with classifying motor skills and the application of different elements of motor learning such as giving instructions of different types and performing practice in a random versus fixed array. |
| Introduction of an illustrated conceptual model of motor learning elements. | The model was used to draw the general layout of the elements that were discussed in the course. Participants were encouraged to use the model framework to link the knowledge they gain into a global perspective of the field. |
| Introduction to and a hands-on practice with a clinical self-directed form. | The form was presented during the didactic module and participants acquired experience applying the conceptual model and using the form during work with their own patients. Each participant was given the opportunity to present his experience and a discussion was made about the challenges and appropriate strategies to overcome the implementation barriers. |
| **Course references** | |
| Textbooks   - Magill, R. A. Motor Learning and Control: Concepts and Applications. 9th Ed. New York, NY: McGraw-Hill; 2010. - Schmidt, R. A. & Lee, T. D. Motor Control and Learning: A behavioral emphasis. Human Kinetics, 5th edition; 2011. - Shumway-Cook, A. & Woollacott, M.H. Motor Control: Theory and Applications: Translating research into clinical practice, 4th Ed. Baltimore, MD: Lippincott, Williams and Wilkins; 2011. | |
