# Supplementary material for: Assessing the impact of a knowledge translation intervention on physical therapists’ self-efficacy and implementation of motor learning practice
Source: BMC Med Educ. 2023 May 23;23:369. doi: 10.1186/s12909-023-04304-2 (PMC10207706; doi:10.1186/s12909-023-04304-2)
Supplement: Supplementary file 2 — Supplementary Material 2 [file 12909_2023_4304_MOESM2_ESM.docx]

**Additional file 2: Structured clinical-thinking form**

#: ____________________

Date: __________________

| **Key element 1: Selection and analysis:** | |
| --- | --- |
| 1.a. Motor skill for practice: ____________________________  1.b. Personal goals related to the chosen skill:   \|  \| \| --- \| \|  \| \|  \| \|  \|   **1.c. Skill classification**  □ gross/ □fine; □ open/ □ close ; □discrete/□ serial/ □ continuous; Gentile's Taxonomy: ________ | |
| **1.d. Specific characteristics of the skill (need to be maintained during practice)**   \| Primary source of information during practice: \| \| --- \| \| □ visual □ auditory □ proprioceptive □ Tactile □ vestibular \| \| Cognitive function involved in practice (e.g. divided attention, decision making ):   - ____________________________________________________________________ - _____________________________________________________________________ \| \| Environmental context in the real world   - ______________________________________________________________________ - ______________________________________________________________________ - ______________________________________________________________________ \|   **1.e. Stage of learning (mark location with X)**  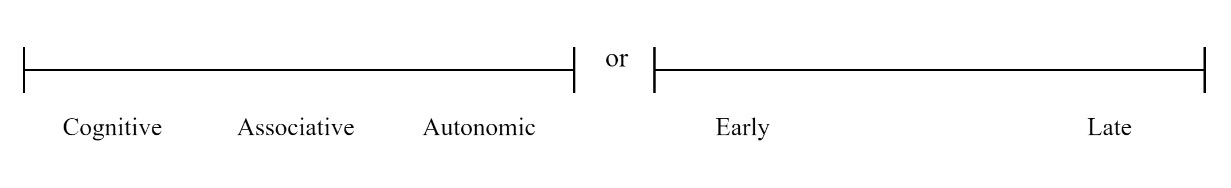  **1.f. Challenging components of the skill for the learner**  □balance; □ active movement and strength; □ sensory processing and integration; □ cognitive and attentional demands; □self-efficacy  **Key element 2: Practice organization**  **2.a. No. of repetitions/duration of practice**   \| Means to enhance practice time (e.g. group, diary, follow-up) \| No. of repetition/ minutes of practice within a session \| Total no. of repetitions /hours of practice \| No. of weeks \| No. of practice sessions per week \| \| --- \| --- \| --- \| --- \| --- \| \|  \|  \|  \|  \|  \| | |
| **2.b. Instructions**   \| Mode of instructions:  □verbal; □demonstrations; □sensory cues; □video ; other ____________________ \| \| --- \| \| Internal focus of attention (movement components)   - ______________________________________________________________________ - ______________________________________________________________________ - ______________________________________________________________________ \| \| External focus of attention (outcome components):   - ______________________________________________________________________ - ______________________________________________________________________ \|   **2.c. Variability of practice**   \| Environmental conditions for practice:   - ______________________________________________________________________ - ______________________________________________________________________ - ______________________________________________________________________ \| \| --- \| \| Variability in motor demands: \| \| □speed; □strength; □distance; □ direction; □postural alignment; other _______________ \| | |
| **2.d. Schedule/order of practice**   \| □blocked ; □serial ; □random \| \| --- \| \| Other skills for random practice   - ______________________________________________________________________ - ______________________________________________________________________ \|   **2.e. Practicing the skill as a whole or as a part**   \| □ Whole  □ part  If part specify the parts to practice:   - ______________________________________________________________________ - ______________________________________________________________________ \| \| --- \| | |
|  | **2.f. Competitive movement strategies**   \| Competitive movement strategies:   - _______________________ \| Measures for restriction of competitive movement strategies:  _________________________________________________ \| \| --- \| --- \| \| - _______________________ \| _________________________________________________ \| \|  \|  \| |
| **2 .g. Feedback**   \| Feedback type \| \| \| --- \| --- \| \| □ Knowledge or results  Outcome measures to give feedback at:   - _________________________________ - _________________________________ \| □ Knowledge of performance  Movement components to give feedback at:   - _________________________________ - _________________________________ \| \| Feedback frequency: □ constant; □ summary; □ faded; □ self-controlled \| \| \| Feedback timing: □ during performance (concurrent); □ terminal \| \| | |
|  | |
| **2.h. Reward (positive reinforcement)**   \| Criterion for reward   - ______________________________________________________________________ - ______________________________________________________________________ \| \| --- \| \| Type of reward:   - ______________________________________________________________________ \| | |

| **key element 3: Assessment of learning outcomes and of the learning process**  **3.a. Outcome variables**  □ reaction time; □ movement time; □ no. of mistakes; □ distance; □ % of successful trials; □ time of keeping balance; other _____________________  **3.b. Standardized assessment tools**   \| Outcome variables:   - ______________________________ - ______________________________ \| Assessment tool:   - ___________________________________ - ___________________________________ \| \| --- \| --- \|   **3.c. Time points for assessment**  □ beginning of practice period; □ end of practice period; □ removed from practice (retention); Other ______________________  **3.d. Learner characteristics which affected the learning process**   \| Barriers   - _______________________________ - _______________________________ - _______________________________ \| Facilitators   - __________________________________ - __________________________________ - __________________________________ \| \| --- \| --- \|   **3.e. Environmental condition which affected the learning process**   \| Barriers   - _______________________________ - _______________________________ - _______________________________ \| Facilitators   - __________________________________ - __________________________________ - __________________________________ \| \| --- \| --- \| |
| --- | --- | --- | --- | --- | --- | --- |
